# Patterns of Multidrug Resistance and Treatment Outcomes Among Pulmonary Tuberculosis Patients in Bangladesh

**DOI:** 10.3390/pathogens15020208

**Published:** 2026-02-12

**Authors:** Naima Nur, Azaz Bin Sharif, Anish Khan, Md Rashedul Islam, Hafid Soualhine, Zubaida Nasreen, Ahmadul Hasan Khan, Pronab Kumar Modak, Mohammad Faridul Alam, Safa Islam, Saeema Islam, Nisha Khan, Meenu Kaushal Sharma

**Affiliations:** 1 Tuberculosis Reference Laboratory, Management Sciences for Health, Gulshan-1, Dhaka 1212, Bangladesh; 2 Department of Public Health, North South University, Bashundhara R/A, Dhaka 1229, Bangladesh; 3 Department of Biotechnology, Chaudhary Ranbir Singh University, Jind 126102, Haryana, India; 4 National Reference Centre for Mycobacteriology, National Microbiology Laboratory, Public Health Agency of Canada, Winnipeg, MB R3E 3R2, Canada; 5 Department of Medical Microbiology, University of Manitoba, Winnipeg, MB R3T 5V6, Canada; 6 National Tuberculosis Control Programme, Mycobacterial Disease Control, Directorate General of Health Services, Mohakhali, Dhaka 1212, Bangladesh; 7 Clinical Research, Sheridan College, Davis Campus 7899, McLaughlin Road, Brampton, ON L6Y 5H9, Canada; 8 School of Life and Medical Science, University of Hertfordshire, Hatfield, Hertfordshire AL10 9AB, UK; 9 Pulse Specialized Hospital Limited, Dania, Kadamtoli, Jatrabari, Dhaka 1236, Bangladesh; 10 Department of Biotechnology, University Institute of Engineering & Technology, Maharshi Dayanand University, Rohtak 124001, Haryana, India

**Keywords:** diagnostics, multidrug-resistant, *Mycobacterium tuberculosis*, pulmonary tuberculosis

## Abstract

**Background:** To effectively manage tuberculosis (TB), it is essential to address the high incidence of the disease, as multidrug-resistant pulmonary TB (MDR-PTB) remains a significant concern to halt pre-extensive drug-resistant (pre-XDR) recrudescence. The objective of the current study was to examine and compare MDR-PTB patterns among adult PTB patients (>12 years) in Bangladesh’s urban and rural areas who had newly diagnosed and previously treated PTB. **Methods:** A total of 430 newly diagnosed and previously treated adult patients with PTB were randomly recruited during two study periods: the 1st period, from May 2010 to December 2010 (eight months), and the 2nd period, from January 2014 to January 2015 (thirteen months). Only the drug-resistant (DR) patients were included in the final analysis. Mycobacteriological tests, i.e., smear microscopy, culture, drug susceptibility testing (proportion method of Canetti), line-probe assay, and GeneXpert MTB/RIF were performed. Logistic regression analysis was used to determine the strength of associations between treatment outcomes and predictor variables. **Results:** Of the newly diagnosed patients, 156 cases were negative and drug-sensitive (DS) at diagnosis, and 274 patients exhibited various DR patterns. During the 1st period, MDR-PTB was 26% among newly diagnosed patients, while the proportion was 31% among previously treated patients in the 2nd period. The majority of MDR-PTB belonged to the age group of ≤45 years. Male patients consistently revealed a higher proportion of MDR-PTB compared to females in both the newly diagnosed and previously treated groups. **Conclusion:** The proportion of MDR-PTB was higher among the previously treated patients than among newly diagnosed patients. Regardless of demographic characteristics, a significant proportion of patients showed DR, particularly in previously treated groups, indicating a substantial burden of MDR-PTB.

## 1. Introduction

Tuberculosis (TB) is a leading cause of death worldwide despite being a preventable and often curable disease. The World Health Organization (WHO) classifies Bangladesh as a high-burden country for TB. In 2019, the Global Burden of Diseases, Injuries, and Risk Factors study estimated that there were approximately 2,16,000 new TB cases and 29,100 deaths among HIV-negative people in the country [1]. Even with universal efforts to fight multidrug-resistant TB (MDR-TB), it is still posing a significant challenge to control TB globally.

MDR-TB is one form of drug-resistant (DR)-TB in which *Mycobacterium tuberculosis (Mtb)* strains are resistant to two first-line anti-TB drugs; rifampicin and isoniazid [2]. Extensive drug resistant (XDR)-TB is MDR/RR-TB and is also resistant to any fluoroquinolones and at least one additional Group A drug (Group A drugs are the most potent group of drugs in the ranking of second-line medicines for the treatment of drug-resistant forms of TB using longer treatment regimens and comprise levofloxacin, moxifloxacin, bedaquiline and linezolid) [3]. Pre-extensive drug-resistant (pre-XDR) is described as MDR-TB with resistance to any fluoroquinolones or injectable second-line drugs [4,5].

Emergence of MDR-TB reflects a profound inadequacy within the global community to address a treatable disease [6,7,8]. Bangladesh is in the 7th position globally for TB, with the cause being high morbidity and mortality [7,9]. Similarly, it is ranked 10th in MDR-PTB out of 27 countries worldwide [7]. According to the Global TB Report-2024, MDR-TB occurred at a frequency of 3.2% among new and 16% among previously treated cases worldwide [10]. In Bangladesh, MDR-TB occurred at a frequency of 1.1% and 5.2% among new and previously treated cases, respectively [10]. According to the recent drug-resistant survey (DRS), 2.3% of new and 13.8% of the previously treated cases in Bangladesh had MDR-TB in 2017 [11]. The United Nations (UN) Sustainable Development Goals placed TB among health targets to end the TB epidemic by 2035, with the target of reducing TB incidence by 90% and TB deaths by 95% [12].

A primary obstacle to reaching these goals is the emergence of MDR pulmonary tuberculosis (PTB), which arises as a result of insufficient and incomplete treatment; patients who stop taking their medicine those who do not follow prescribed treatment consistently, poor drug quality, ensuring an appropriate storage environment at all times, poor treatment adherence (as it is challenging to maintain a single provider for each MDR patient consistently), and availability of rapid diagnostics. According to patients, available equipment and facilities are inadequate, and there is inadequate training for healthcare professionals [11,12,13]. Delayed diagnosis may also result in wider disease transmission, worse disease progression, and significant adverse outcomes, including increased risk of death. Additionally, a significant number of TB patients are directly infected with genetically resistant strains of *Mtb*, which can lead to the development of further drug-resistance and the emergence of MDR-PTB or resistant forms of MDR-PTB (i.e., pre-XDR-PTB) [14,15]. Furthermore, nosocomial transmission is another source of DR-TB infection during treatment [16]. Ensuring proper and early identification of cases and pleasing treatment outcomes is vital to limit TB transmission, minimize drug resistance risk, and control TB disease [15,17]. Treatment for MDR-PTB requires second-line TB drugs that are expensive, time-consuming, and have unfavorable clinical outcomes, including significant adverse pharmacological effects, that require monitoring and counseling. Numerous trials have suggested that the therapeutic success rate of such treatment is only 60% to 70% [6,18,19]. In Bangladesh, >70% of MDR-PTB cases are successfully treated [20]. According to Public Health Statistics, the cost of treating DR-TB is 20-fold higher than that of drug-susceptible (DS) TB [18]. The WHO recommended genotypic drug susceptibility testing (DST), a rapid and reliable molecular diagnostic test or probe-based assay diagnosis for patients at risk of MDR-PTB [19]. When TB is diagnosed for MDR, a standard line probe assay (LPA) such as GenoType MTBDRplus can detect *rpoB* gene mutation for rifampicin and *katG* and *inhA* gene mutation for isoniazid [18,21]. GeneXpert MTB/RIF, also referred to as GeneXpert [22] and various formats [22,23], that also detects *rpoB* (Rv0664) gene mutation for rifampicin, but GeneXpert assay may not be conclusive in detecting MDR-PTB based on rifampicin resistance [23,24].

Although rapid advancement of molecular diagnostic techniques has made it possible to identify *Mtb* quickly, control of TB disease has become increasingly challenging due to MDR, particularly in developing countries like Bangladesh. Moreover, very little is known about patterns of MDR-TB among newly diagnosed patients who completed a six-month first-line drug treatment regimen, and previously treated patients who failed initial therapy, experienced relapse after treatment completion or defaulted after at least two months of initial treatment, with PTB in Bangladesh. Therefore, the current investigation aimed to determine patterns of MDR-PTB in Bangladesh.

## 2. Materials and Methods

### 2.1. Ethics Statement and Informed Consent Statement

The study received ethical approval from the North South University Ethical Review Committee/Institutional Review Board, Dhaka, Bangladesh (2025/OR-NSU/IRB-No. 0261). It was performed in accordance with the Declaration of Helsinki. The National Tuberculosis Control Programme, Bangladesh, approved the study for publication of the current manuscript *vide* Letter No. 4375 dated 12 August 2025. Participants were enrolled in the study only after providing informed written consent.

### 2.2. Inclusion and Exclusion Criteria

PTB patients were categorized into newly diagnosed patients, who completed a six-month first-line drug treatment regimen, and previously treated patients, who failed initial therapy, experienced relapse after treatment completion or defaulted after at least two months of initial treatment. These groups use their drug history records from record keeping (TB Manager). They were HIV-negative, both male and female genders, non-pregnant women, and >12 years old. They received PTB treatment at the National Institute of Diseases of the Chest Hospital (NIDCH) in two study periods and provided written consent before collecting samples. After diagnosis, patients with negative results and patients with drug-sensitive isolates were excluded from the current study.

### 2.3. Study Samples

**Figure 1:**
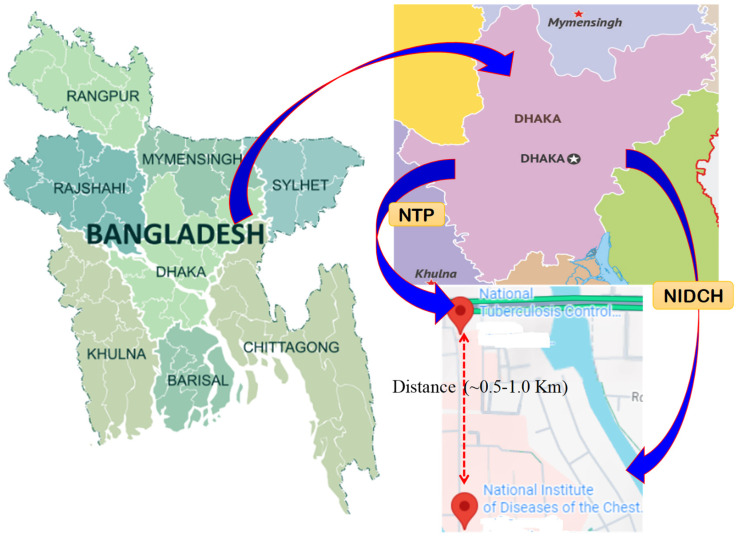
A graphical location of National Institute of Diseases of the Chest & Hospital (NIDCH) and National Tuberculosis Programme (NTP), Dhaka, Bangladesh. The distance between NTP and NIDCH (~0.5–1 km) is tiny compared to the national scale.

A cross-sectional study was conducted using a quantitative approach. Four hundred thirty (n = 430) newly diagnosed and previously treated patients were admitted for treatment at NIDCH. Only DR patients were included in the final analysis. This hospital is the only tertiary referral hospital for TB and chest diseases in Dhaka, Bangladesh (Figure 1), and receives patients from the entire country, as it is a national hospital. Furthermore, patients who are suspected of having TB or those already being treated, as well as those being re-treated, are visited regularly in the hospital. However, our study focuses on treating and re-treating patients who are initiating Anti-Tubercular Treatment (ATT).

**Figure 2:**
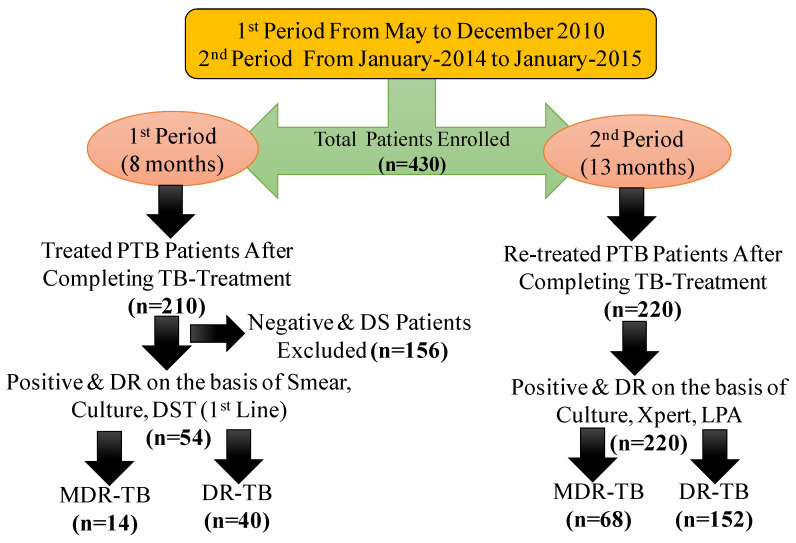
Flowchart for categorizing participants with their MDR patterns in two different study periods. Note: PTB = Pulmonary TB, LPA = Line Probe Assay, DST = Drug Susceptibility Testing, DR = Drug Resistant, MDR = Multi-Drug Resistant, DS = Drug Sensitive.

The study was divided into two periods. We collected sputum samples of patients up to 8th months (from May 2010 to December 2010) as well as 13th months (from January 2014 to January 2015) from the hospital regularly (Figure 2). Demographic information, including gender and age, as well as mycobacteriological tests, were recorded from the enrolled participants during both periods. The sample size was calculated using a formula for proportions from a previous study [8]. Assuming the percentage of MDR-PTB patients will be 25% in this tertiary-level hospital, the formula of proportions is:

n = z^2^ (pq)/d^2^ which equals to
288.12 (~289).
Here,
n = Desired sample size
z = 95% confidence level usual value is 1.96
p = Proportion of target population (0.25)
q = (1 − p)
d = Allowable error (0.05)

In the 1st study period, a total of 210 newly diagnosed PTB patients who completed a six-month first-line drug treatment regimen were obtained. The patients (144 males and 66 females, aged between 15 and 75 years) received their diagnoses using conventional, acid-fast smear testing conducted with light and fluorescence microscopy. The identification was made on culture-positive *Mtb* isolates, which then underwent DST for first-line drugs. In the 2nd study period, a total of 220 previously treated PTB patients, including those who failed initial therapy, experienced relapse after treatment completion or defaulted after at least two months of initial treatment, were collected. The patients (146 males and 74 females, aged between 15 and 75 years) received their diagnoses using conventional culture, GeneXpert MTB/RIF, and LPA (GenoTypeMTBDRplus).

### 2.4. TB Identification and Diagnostic Confirmation

The collected sputum specimens were decontaminated and inoculated on Lowenstein–Jensen slants for culture using a conventional method of Petroff [25]. Concentrated smears were visualized after staining with the Ziehl–Neelsen method [26] and the auramine O staining method [27]. TB-positive samples were tested for the presence of *Mtb*, since TB-negative samples cannot be identified as *Mtb*, which was excluded from the study. *Mtb* was identified by multiple identification tests, including biochemical tests, i.e., nitrate reduction and catalase assays, and sensitivity test i.e., p-nitrobenzoic acid (PNB). PNB was also performed, which is reported to be a specific inhibitor of *Mtb* [28]. All identification tests were performed manually, but molecular identification tests were done automatically.

### 2.5. Culture-Based Phenotypic DST and Molecular-Based Genotypic DST

Phenotypic DST of the identified isolates was examined by the proportion method of Canetti using first-line drugs isoniazid (0.2 μg/mL), rifampicin (40 μg/mL), ethambutol (2 μg/mL), and streptomycin (4 μg/mL) [29]. Pyrazinamide was not tested due to technical challenges with the drug for phenotypic susceptibility testing [30]. DR-PTB diagnosis has been carried out by using molecular-based genotypic DST assays, i.e., GeneXpert MTB/RIF (Cepheid, Sunnyvale, CA, USA) [23] and LPAs (GenoTypeMTBDRplus-Hain LifeScience GmbH, Nehren, Germany) [21].

### 2.6. Statistical Analysis

Data were analyzed using STATA statistical software (StataCorp. 2021. Stata: Statistical Software: Release 16. College Station, TX, USA: StataCorp LLC). Descriptive analysis was performed using frequency and percentage. The Chi-square test examined the association between the dependent and independent variables. *p*-values < 0.05 were considered statistically significant. Logistic regression was used to analyze the strength of associations between outcome and predictor variables. Odds ratios with a 95% confidence interval were calculated.

## 3. Results

### 3.1. Study Participants and Drug Resistance Patterns

A total of 430 PTB patients (210 newly diagnosed and 220 previously treated) were diagnosed. Among newly diagnosed PTB patients, 148 patients were negative and recovered, while 8 patients had DS. Negative and DS patients were excluded from the study. Final analysis included 274 patients with varying DR patterns (Figure 2). As described in Table 1, in the 1st period, 62 out of 210 newly diagnosed PTB patients tested positive for *Mtb* by culture. Out of the 62 (30%) isolates, 60 (29%) were smear-positive by both microscopic techniques, i.e., light microscopy and fluorescence microscopy, and 62 (30%) patients were identified to be positive through identification tests. Of the 62 patients, 8 (13%) were susceptible to all first-line TB drugs tested, and the remaining patients showed variable resistance patterns. Among 54 patients (87%), inconsistent resistance patterns were found as follows: mono-drug resistance in 2 patients (3.7%), MDR (i.e., resistance to both rifampicin and isoniazid among first-line drugs) in 14 patients (26%), and polydrug resistance (i.e., resistance to more than one first-line drug, but not both rifampicin and isoniazid together) was found in 38 patients (70%). The frequency of MDR was then determined to measure the proportion of MDR-PTB. Any resistance refers to total resistance. Resistance to isoniazid was observed in 42 (78%), rifampicin in 24 (44%), ethambutol in 32 (59%), and streptomycin in 40 (74%) isolates (Table 2). Among the 220 previously treated PTB patients, 68 (31%) were MDR-PTB, and the remaining patients were DR-PTB in the 2nd period. Rifampicin resistance was detected in 106 (48.2%) isolates by both LPA and GeneXpert MTB/RIF, and isoniazid resistance was found in 182 isolates (82.7%), as described in Table 1. The proportion of MDR-TB was 26% among newly diagnosed patients and 31% among previously treated patients. Patients with DS isolates who received first-line medications were exclusively identified during the initial phase of the study, as they had not encountered drug pressures from prior treatments yet.

### 3.2. Different Genders and Ages in Drug Resistance Patterns

Demographic information was compared within drug patterns in the newly diagnosed and previously treated patients. Most patients aged between 15 and 75 belonged to the reproductive age group (15–45 years). Male patients had 68% positivity in the 1st period and 67% in the 2nd period compared to females. Within male patients, MDR-PTB was 75% in newly diagnosed and 72% in previously treated patients. However, there were no statistically significant differences (*p* > 0.05). In logistic regression analysis within drug resistance patterns, after adjusting for the factors, sociodemographic parameters were not statistically associated (Table 3).

## 4. Discussion

The current study was unique in that it identified MDR-PTB among both newly diagnosed and previously treated individuals. Throughout the two study periods, DR-PTB isolates showed variable resistance patterns to first-line anti-TB drugs. Among patients diagnosed with MDR-PTB, 26% were from the newly diagnosed group, while a higher proportion (31%) was observed among previously treated patients. Similar results on MDR-PTB were also reported by several researchers in Bhutan [31], Ethiopia [13], and Pakistan [32]. Similarly, Bangladesh’s 2010–2011 first nationwide DRS showed MDR-TB prevalence of 28.5% in previously treated patients [33], but it was lower in Bangladesh’s 2014 sentinel surveillance DRS (13.8%) [11], and higher than that reported in China (44%) [34].

Unlike other studies [11,33], mono resistance was lower, and polydrug resistance was higher in the current study. Among the first-line anti-TB drugs, total resistance to isoniazid was the highest in first-line drugs, and this result is consistent with a previous study in China [34]. Total isoniazid resistance in previously treated patients was higher than that reported in Iran (47%), China (53.2%), and Pakistan (37.1%) [32,35]. Among newly diagnosed patients, total streptomycin and ethambutol resistance in the present study was higher than that described in Pakistan [32] and China [34]. Similarly, total rifampicin resistance in previously treated patients was higher than that reported in Pakistan (25.2%) and Iran (38.2%) but was lower than the resistance (54.3%) observed by Kundu et al. in Bangladesh [32,35]. We found relatively fewer cases of rifampicin resistance in newly diagnosed patients compared to the previous study [24].

The study found no statistical significance (*p* > 0.05) between patient demographics and drug patterns in the newly diagnosed and previously treated patients. However, most of the MDR patients belonged to the reproductive age group. Male patients consistently revealed higher proportions of drug resistance, which included MDR-PTB, compared to females in the two study periods. Previous studies demonstrated a similar outcome when studying newly diagnosed and previously treated patients [32,34].

Our research emphasizes the importance of understanding resistance patterns in treating PTB. It highlights the necessity of ensuring patient adherence to treatment, as well as the need for the design and implementation of nationwide preventive strategies aimed at reducing transmission rates of TB relapse rates and minimizing drug resistance to first-line drugs. The following three recommendations are made based on the above findings and should shed light on further intervention and research: (i) it is essential to educate the medical staff at the clinic level, for non-government organizations in the community, for hospital staff, and other disciplinary team members at the hospital, and social workers in the health care system, (ii) it is also crucial to make awareness to the patients and their families on DR-TB, because a patient’s recovery depends on the family’s ability to create a safe, nurturing, supportive environment, and at the same time the government, community, and health care providers also play a significant role, and (iii) implement better and faster identification of DR-TB and introduce newer MDR regimens, XDR-TB, as WHO recommended (i.e., BPaL) [36]. Although the Bangladesh National Tuberculosis Control Programme is implementing its nationwide TB control strategies, the high prevalence of DR-PTB poses a real challenge. It is essential to take appropriate action immediately to stop the rise in drug resistance. Screening a larger volume of TB patients attending TB hospitals and clinics is necessary, and the patients should be evaluated regularly by specialized clinicians during the continuous phase of anti-TB treatment. At the same time, preventive measures should be taken appropriately to prevent the transmission of MDR-TB in the community [4]. It is also crucial to develop capacity not only for genotypic DST but also for whole-genome sequencing technologies and bioinformatics analysis in resource-limited settings. Psychological distress in PTB patients is common due to the long treatment duration. The development of nationwide psychological counseling programs is needed, as described by Nur et al. in Bangladesh [8]. All of the measures mentioned above are critical to stopping the spread of MDR-TB, improving treatment outcomes, and playing a positive role in the effort to control and eliminate DR-TB. The national policymakers, program managers, and international stakeholders should pay more attention to MDR-TB to halt pre-XDR and XDR recrudescence. To achieve the WHO and UN goals of eradicating TB by 2035, gaps in diagnostic, preventative, and therapeutic activities must be filled. Therefore, further research, including a nationwide study, is needed to elucidate the MDR-PTB rate in newly diagnosed and previously treated patients. Our findings would undoubtedly spark interest among national and international communities of researchers and guide policymakers in developing specific recovery and rehabilitation plans, programs, and policies for PTB affected people.

The present study represents, to our knowledge, the inaugural cross-sectional investigation into the patterns of MDR-PTB among both newly diagnosed and previously treated patients with PTB in Bangladesh. We assessed the drug-resistance patterns of *Mtb* in PTB patients, encompassing individuals who underwent newly diagnosed and previously treated during two distinct timeframes. However, the sampling approach was not exactly the same for both phases of the study. Due to limited resources, hospital policies and standards over different time periods and time constraints, we could not use the same laboratory methods in both study periods.

## 5. Conclusions

The study revealed that drug resistance patterns of *Mtb* have become ever more challenging in developing countries. The proportion of MDR-PTB was found to be higher among patients who had undergone retreatment compared to those receiving initial treatment, emphasizing close monitoring of drug therapy, drug compliance, and development of drug resistance while the patient is on initial treatment. It highlights the critical need for the implementation of robust control measures and the enhancement of treatment strategies to address the issue of DR-TB effectively.

## Figures and Tables

**Table 1:** Positive rate of Paraclinical data in two study periods.

No. of Participants & Duration	Treatment Type	Culture	Microscopy		Phenotypic DST		Genotypic DST				
(n = 210) & 1st Period	Newly diagnosed	LJ n (%)	LM n (%)	FM n (%)	DS n (%)	DR n (%)	Xpert RIF ^R^ n (%)	Xpert RIF ^S^ n (%)	LPA INH ^S^ & RIF ^R^ n (%)	LPA INH ^R^ & RIF ^S^ n (%)	LPA INH ^R^ & RIF ^R^ n (%)
		62 (30)	60 (29)	60 (29)	8 (13)	54 (87)	-	-	-	-	-
(n = 220) & 2nd Period	Previously treated	220 (100)	-	-	-	-	106 (48)	114 (52)	38 (17)	114 (52)	68 (31)

Note: LJ = Lowenstein-Jensen; LM = Light Microscopy; FM = Fluorescence Microscopy; DR = Drug Resistant; DST = Drug Susceptibility Testing; LPA = Line Probe Assay; n = number of patients; RIF = Rifampicin; INH = Isoniazid; R = Resistant; S = Sensitive.

**Table 2:** Drug resistance patterns of patients (n = 54) in the 1st study period.

Resistance to First-Line Anti-TB Drug		Frequency (%)
Any resistance to	INH	42 (78)
	RIF	24 (44)
	EMB	32 (59)
	SM	40 (74)
Mono-drug resistance to	SM	2 (3.7)
Multidrug resistance to	INH + RIF	10 (18.5)
	INH + RIF + EMB	4 (7.4)
Polydrug resistance to	RIF + SM	10 (18.5)
	INH + EMB + SM	28 (52)

Note: INH = Isoniazid; RIF = Rifampicin; EMB = Ethambutol; SM = Streptomycin; Any resistance (resistance to Mono-drug + Multidrug + Polydrug).

**Table 3:** Patients’ characteristics with drug patterns in two different study periods.

Variables	1st Study Period (Between May to December 2010)					2nd Study Period (Between January 2014 to January 2015)				
	DR (n = 40) F (%)	MDR (n = 14) F (%)	Total (n = 54) F (%)	*p*-Value (Chi-Square)	Adjusted OR (95%CI)	DR (n = 152) F (%)	MDR (n = 68) F (%)	Total (n = 220) F (%)	*p*-Value (Chi-Square)	Adjusted OR (95%CI)
Age				0.636					0.477	
15–45	32 (80)	12 (86)	44 (81)		Reference	114 (75)	54 (79)	168 (76)		Reference
46–75	8 (20)	2 (14)	10 (19)		0.63 (0.11 to 3.61)	38 (25)	14 (21)	52 (24)		0.74 (0.37 to 1.49)
Gender	0.920					0.312				
Female	12 (30)	4 (29)	16 (30)		Reference	53 (35)	19 (28)	72 (33)		Reference
Male	28 (70)	10 (71)	38 (70)		1.2 (0.30 to 4.85)	99 (65)	49 (72)	148 (67)		1.4 (0.76 to 2.70)

Note: DR = Drug Resistant, MDR = Multi-Drug Resistant, F = Frequency, OR = Odds Ratio, CI = Confidence Interval, n = number of patients; Reference category is coded as 1.00.

## Data Availability

There are no raw data generated in this study.

## Abbreviations

Abbreviations	Full Names	Definition/Criteria
TB	Tuberculosis	TB is the name of a disease caused by *Mtb*
*Mtb*	*Mycobacterium tuberculosis*	*Mtb* is the causative agent of TB
MDR	Multidrug resistant	Resistance to both rifampicin and isoniazid among first-line drugs
DR	Drug resistant	*Mtb* does not respond to any drugs
pre-XDR	Pre-extensive drug-resistant	pre-XDR against any fluoroquinolones or second-line drugs
RR	Rifampicin-Resistant	MDR-TB is resistant to RR
XDR-TB	Extensive drug-resistant	XDR-TB is resistant to any fluoroquinolones and at least one additional second-line drug
DS	Drug-sensitive	Mtb sense to against standard drugs
PNB	p-nitrobenzoic acid	PNB is a specific inhibitor of *Mtb*
DRS	Drug-resistant survey	DRS is a survey of drug resistance in individuals
WHO	World Health Organization	WHO is repute health organization
DST	Drug susceptibility testing	DST is a method of drug testing against *Mtb*
LPA	Line probe assay	It is a *Mtb* detection assay
ATT	Anti-Tubercular Treatment	It is the treatment of TB
UN	United Nations	The UN is an international organization
NIDCH	National Institute of Diseases of the Chest Hospital	NIDCH is a national government hospital in Dhaka, Bangladesh
NTP	National Tuberculosis Programme	NTP is a national programme of Dhaka, Bangladesh
